# Use of big data in the surveillance of veterinary diseases: early detection of tick paralysis in companion animals

**DOI:** 10.1186/s13071-016-1590-6

**Published:** 2016-05-23

**Authors:** Vanina Guernier, Gabriel J. Milinovich, Marcos Antonio Bezerra Santos, Mark Haworth, Glen Coleman, Ricardo J. Soares Magalhaes

**Affiliations:** School of Veterinary Science, The University of Queensland, Gatton Campus, Gatton, 4343 QLD Australia; Federal Rural University of Pernambuco, Academic Unit of Garanhuns, Garanhuns, 55292-270 PE Brazil; Child Health Research Centre, The University of Queensland, Herston, 4006 QLD Australia

**Keywords:** Australia, Companion animals, Digital epidemiology, Dogs and cats, Google, Google Trends, Internet, Notified cases, Syndromic surveillance, Tick paralysis

## Abstract

**Background:**

Tick paralysis, resultant from envenomation by the scrub-tick *Ixodes holocyclus*, is a serious threat for small companion animals in the eastern coast of Australia. We hypothesise that surveillance systems that are built on Internet search queries may provide a more timely indication of high-risk periods more effectively than current approaches.

**Methods:**

Monthly tick paralysis notifications in dogs and cats across Australia and the states of Queensland (QLD) and New South Wales (NSW) were retrieved from *Disease WatchDog* surveillance system for the period 2011–2013. Internet search terms related to tick paralysis in small companion animals were identified using Google Correlate, and corresponding search frequency metrics were downloaded from Google Trends. Spearman’s rank correlations and time series cross correlations were performed to assess which Google search terms lead or are synchronous with tick paralysis notifications.

**Results:**

Metrics data were available for 24 relevant search terms at national level, 16 for QLD and 18 for NSW, and they were all significantly correlated with tick paralysis notifications (*P* < 0.05). Among those terms, 70.8, 56.3 and 50 % showed strong Spearman’s correlations, at national level, for QLD, and for NSW respectively, and cross correlation analyses identified searches which lead notifications at national or state levels.

**Conclusion:**

This study demonstrates that Internet search metrics can be used to monitor the occurrence of tick paralysis in companion animals, which would facilitate early detection of high-risk periods for tick paralysis cases. This study constitutes the first application of the rapidly emerging field of Internet-based surveillance to veterinary science.

## Background

Tick paralysis is a potentially fatal neurological condition affecting companion animals, livestock and humans. It occurs globally, but the majority of cases are restricted to the Pacific Northwest regions of North America and the eastern coast of Australia [1]. The disease is resultant from exposure to a toxin present in the saliva of paralysis tick species [2–4]. About 69 species of ticks from around the world are capable of inducing paralysis [4], and *Ixodes holocyclus* is the main species in Australia [1, 2, 5]. Annually, *Ix. holocyclus* causes tick paralysis in about 10,000 companion animals [6, 7]. It is considered highly toxic, with one female able to kill a cat or a dog [8]. In Australia, the majority of tick paralysis cases occur between July and December [2], with adult ticks number peaking during spring and early summer [5, 6, 9]. In companion animals, tick paralysis may exhibit a progressive generalised paresis leading to respiratory failure in conjunction with pulmonary oedema and aspiration pneumonia [10, 11]. If left untreated, death can occur within 24 to 48 h of onset of clinical signs [11–13].

Given the acute clinical presentation of the disease and the potentially fatal outcome, reliable surveillance data for the early warning of the occurrence of high-risk periods is essential for the timely preparedness of primary care services. Sentinel surveillance approaches have been used for surveillance of a number of companion animal diseases in Australia [14]. Tick paralysis is among the diseases monitored by the *Disease WatchDog* surveillance system, a prospective national disease surveillance project held in Australia, focusing on dogs and cats [14]. Data are collected from practicing veterinarians as cases of diseases are diagnosed, providing monthly online updates.

In recent years, an emerging field of research has focused on the development of surveillance approaches that utilise various Internet-based data streams to analyse queries from the Internet search engines to predict high-risk periods [15–17]. This approach works on the premise that people who contract a disease are likely to actively seek for information on the symptoms and/or disease on the Internet, and that reliable and timely estimates of diseases activity in the community can be produced by monitoring the frequency of specific Internet search terms [16]. This approach has been documented by numerous studies and produced useful tools for human communicable disease surveillance that work in near real-time and assist the allocation of public health interventions [15, 16, 18, 19].

Recent research has suggested that Internet-based surveillance systems have wider epidemiological applicability for human infectious disease surveillance than has previously been recognised [16]. However, there is no research reporting the use of this approach in veterinary medicine. The present study aims to examine the utility of digital epidemiological approaches based on Internet searches as a means of surveillance of tick paralysis in companion animals in Australia. The specific objectives are twofold: (i) to investigate the statistical correlation between tick paralysis notifications from endemic regions in Australia and search term metrics extracted from Google Trends; and (ii) to demonstrate the utility of digital surveillance systems, specifically systems built on Internet search metrics, as tools for use in veterinary science.

## Methods

### Tick paralysis data

Data on tick paralysis cases used in this study were extracted from *Disease WatchDog* surveillance website (http://www.diseasewatchdog.org). Information available on the *Disease WatchDog* site includes suburb level disease incidence data and monthly updates for registered users [14]. Monthly reports of tick paralysis cases were obtained for a three-year period, spanning 01 January 2011 to 31 December 2013; we focused our analysis on data from this period as it represented the time period with more reliable data series. The reasons behind this decision are two-fold. First, from 01 January 2011 Google Trends data have undergone an improvement to geographical assignment. As the disease watchdog started reporting from September 2010, we decided to discard the 2010 data (only 4 months’ worth of data) to ensure we only used the best from Google. Secondly, upon downloading data from *Disease WatchDog* website we noted that reporting of tick paralysis from January 2014 to the database became inconsistent compared to previous years and we decided to utilise data up until December 2013 to ensure we only used the best from *Disease WatchDog*. Monthly tick paralysis cases were considered only for Queensland (QLD) and New South Wales (NSW) as these two states account for the vast majority of cases reported in Australia (98 %).

### Google search term selection and collection of search metric data

We analysed the words (i.e. search terms) that people used in their searches of the World Wide Web when seeking information on tick paralysis. Counts (i.e. metrics) of these search terms were taken from the Google Trends website (www.google.com/trends). Search metrics are provided as normalised time series; all data are presented as values ranging from 0 to 100, with 100 representing the time point with the highest value (during which the most searches were performed) and other points scaled accordingly. Methods for the identification of relevant search terms, collection of data and the use of these data have previously been described [16]. Briefly, search terms of interest were identified using a two-step approach. Firstly, time series of paralysis tick notifications downloaded from the *Disease WatchDog* website were used to query Google Correlate (www.google.com/trends/correlate). This allows identifying up to 100 search terms with the highest correlation to the three queried time series (i.e. time series related to Australia, QLD, and NSW). Second, search metrics for to the final list of terms were downloaded from the Google Trends website; metrics were collected at national level and at state level for QLD and NSW. Whilst Google Trends provides a function to download.csv text files of search metrics, the level of data aggregation (daily, weekly, monthly) cannot be specified by the user [16]. As such, the required data was “scraped” from Google Trends website using a previously developed script, allowing the problems associated with the level of aggregation to be overcome [16].

### Statistical analyses

Spearman’s rank correlations were performed between monthly notifications of tick paralysis cases submitted to the *Disease WatchDog* website and Google Trends search metrics for all terms with available data; these were performed at both state and national level, allowing the search metrics with the highest degree of correlation with notification data to be identified. A strong correlation was defined as 0.60 ≤ Spearman’s rho < 0.80 and a very strong correlation was defined as rho ≥ 0.80. Bonferroni correction was applied to significance levels to account for multiple testing. All reported *P*-values in this document correspond to one-tailed tests.

Linear associations between Google search metrics and tick paralysis notifications were assessed using cross correlations. Cross-correlations were conducted for Google search terms that exhibited strong or very strong correlations (rho ≥ 0.60) with tick paralysis notifications for Australia, QLD and NSW separately. Cross-correlations were performed with lag values ranging from −7 to +7 using IBM SPSS version 21 software [20]. Cross-correlations should be interpreted as product-moment correlations between search metrics and tick paralysis notifications. This approach allows dependence to be identified between the two time series with respect to temporal offsets, referred to as lags, and to visually judge whether one series tends to lead or lag the other. A lag value of +1 describes correlations that have been performed using Google search data that has been shifted forward (i.e. occurred after tick paralysis notifications) one unit (i.e. one month); conversely, a negative lag indicates that the Google search data has been shifted backwards by the number of units denoted, and thereby occurred before tick paralysis notifications data. Within the context of this work, cross-correlations with significant negative lag values are of most interest as they demonstrate that Google search metrics data are (i) significantly associated with the tick paralysis notifications and (ii) lead the clinical notification by a number of months. Synchronous cross-correlations (i.e. significant positive cross-correlation at lag 0) are also of interest as they demonstrate Google search terms that are coincident with tick paralysis reports.

## Results

### Tick paralysis notifications and Google trends data

**Fig. 1:**
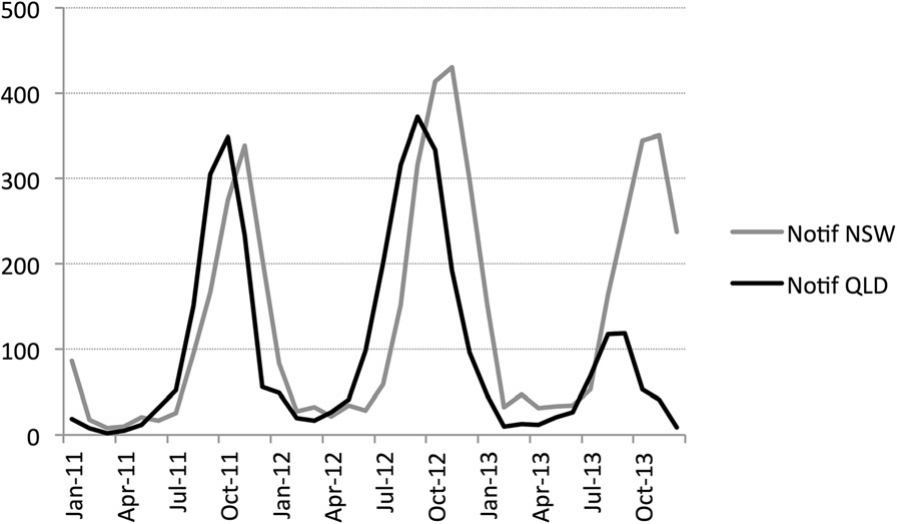
Tick paralysis cases notified on the *Disease WatchDog* website. Monthly notifications originating in the states of New South Wales or Queensland, 2011–2013

Between 1st January 2011 and 31st December 2013, a total number of 8,414 tick paralysis cases were registered nationally in the *Disease WatchDog* website in both canines and felines; a total of 4,891 (58 %) cases were reported as originating in NSW and 3,523 (42 %) in QLD (Fig. 1). The figure indicates that each year the rise and peak of tick paralysis notifications in QLD occurs between September-October while in NSW it occurs in October-November. This difference is statistically significant, cross-correlations analyses between the two states time series showing that the peak of notifications in QLD leads the peak in NSW by one month. In total, 31 search terms were identified as having potential relevance to tick paralysis (Table 1). Search metrics associated to these terms were downloaded from the Google Trends website; they were available for 24 of the 31 identified terms for Australia, 16 for QLD and 18 for NSW.

**Table 1:** Spearman’s rho rank for the search terms related to tick paralysis in Google Trends

	Australia		QLD		NSW	
Search term	rho	*P*-value	rho	*P*-value	rho	*P*-value
Paralysis tick	0.95^a^	< 0.01	0.81^a^	< 0.01	0.89^a^	< 0.01
Tick paralysis	0.91^a^	< 0.01	0.92^a^	< 0.01	0.89^a^	< 0.01
Ticks	0.83^a^	< 0.01	0.82^a^	< 0.01	0.83^a^	< 0.01
Tick	0.81^a^	< 0.01	0.83^a^	< 0.01	0.87^a^	< 0.01
Paralysis ticks	0.85^a^	< 0.01	0.68^b^	< 0.01	0.66^b^	< 0.01
Dog tick	0.76^b^	< 0.01	0.59	< 0.01	0.80^a^	< 0.01
Dogs ticks	0.72^b^	< 0.01	0.88^a^	< 0.01	0.70^b^	< 0.01
Removing ticks	0.70^b^	< 0.01	0.65^b^	< 0.01	0.65^b^	< 0.01
Dog ticks	0.67^b^	< 0.01	0.40	0.016	0.60^b^	< 0.01
Remove tick	0.78^b^	< 0.01	0.78^b^	< 0.01	0.52	< 0.01
Cat tick	0.75^b^	< 0.01	0.72^b^	< 0.01	0.42	< 0.01
Paralysis tick dogs	0.79^b^	< 0.01	0.40	0.015	0.37	0.020
Dog paralysis tick	0.79^b^	< 0.01	0.45	< 0.01	0.43	< 0.01
Paralysis tick dog	0.71^b^	< 0.01	0.37	0.026	0.44	< 0.01
Cats ticks	0.70^b^	< 0.01	0.40	0.015	0.38	0.020
Tick on dog	0.61^b^	< 0.01	0.36	0.026	0.34	0.037
Paralysis tick symptoms	0.77^b^	< 0.01	No data		0.44	< 0.01
Tick in dog	0.58	< 0.01	No data		0.43	< 0.01
Paralysis tick treatment	0.48	0.030	No data		No data	
Tick paralysis in dogs	0.46	< 0.01	No data		No data	
Tick symptoms dog	0.43	< 0.01	No data		No data	
Paralysis tick humans	0.39	0.018	No data		No data	
Dog tick treatment	0.36	< 0.01	No data		No data	
Paralysis tick Australia	0.36	0.030	No data		No data	
Brown dog tick	No data		No data		No data	
Dog tick removal	No data		No data		No data	
Paralysis tick cat	No data		No data		No data	
Paralysis tick cats	No data		No data		No data	
Tick paralysis in humans	No data		No data		No data	
Tick paralysis in cats	No data		No data		No data	
Ticks and dog paralysis	No data		No data		No data	

^a^Top ranked terms that have very strong Spearman’s correlation with the *Disease WatchDog* data (rho ≥ 0.8)

^b^Terms that have strong Spearman’s correlation (rho ≥ 0.6); other terms are correlated in a lesser degree. *P*-values for all search terms with data available were *P* < 0.05, which are represented beside each column

No data, the metrics for that term were not available

### Correlations between Google search terms and tick paralysis cases

**Fig. 2:**
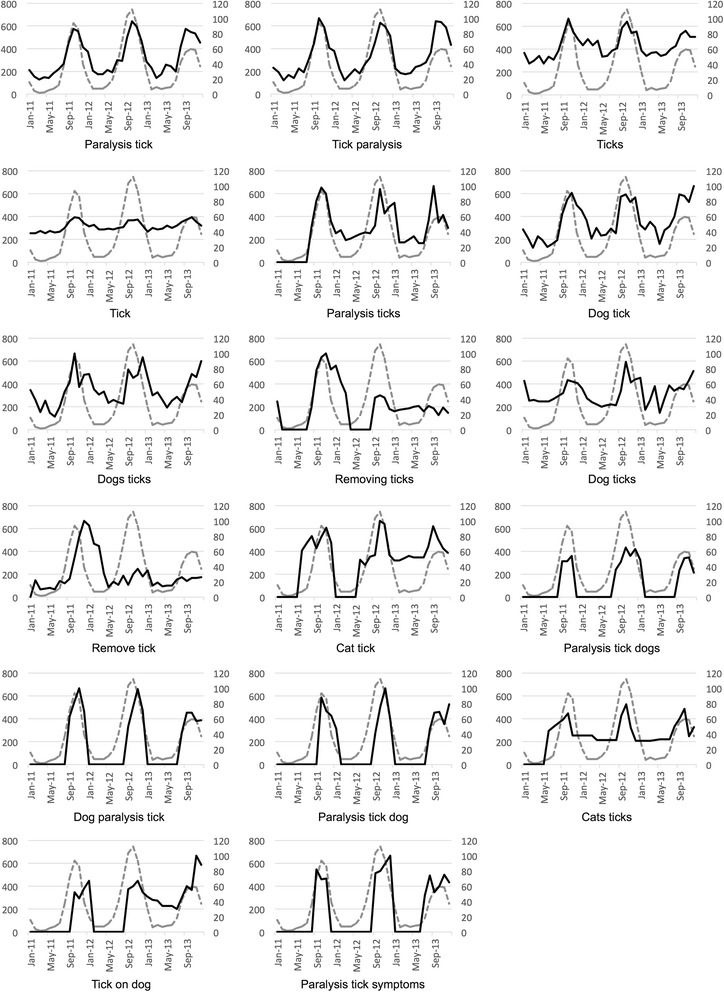
Spearman’s top ranked search terms at the national level. Gray dotted lines show the monthly notifications of tick paralysis cases in Australia, while black lines show the metrics for each of the 17 search terms (on 24) that were ranked as having strongly and very strongly significant Spearman’s correlations (rho ≥ 0.6)

**Fig. 3:**
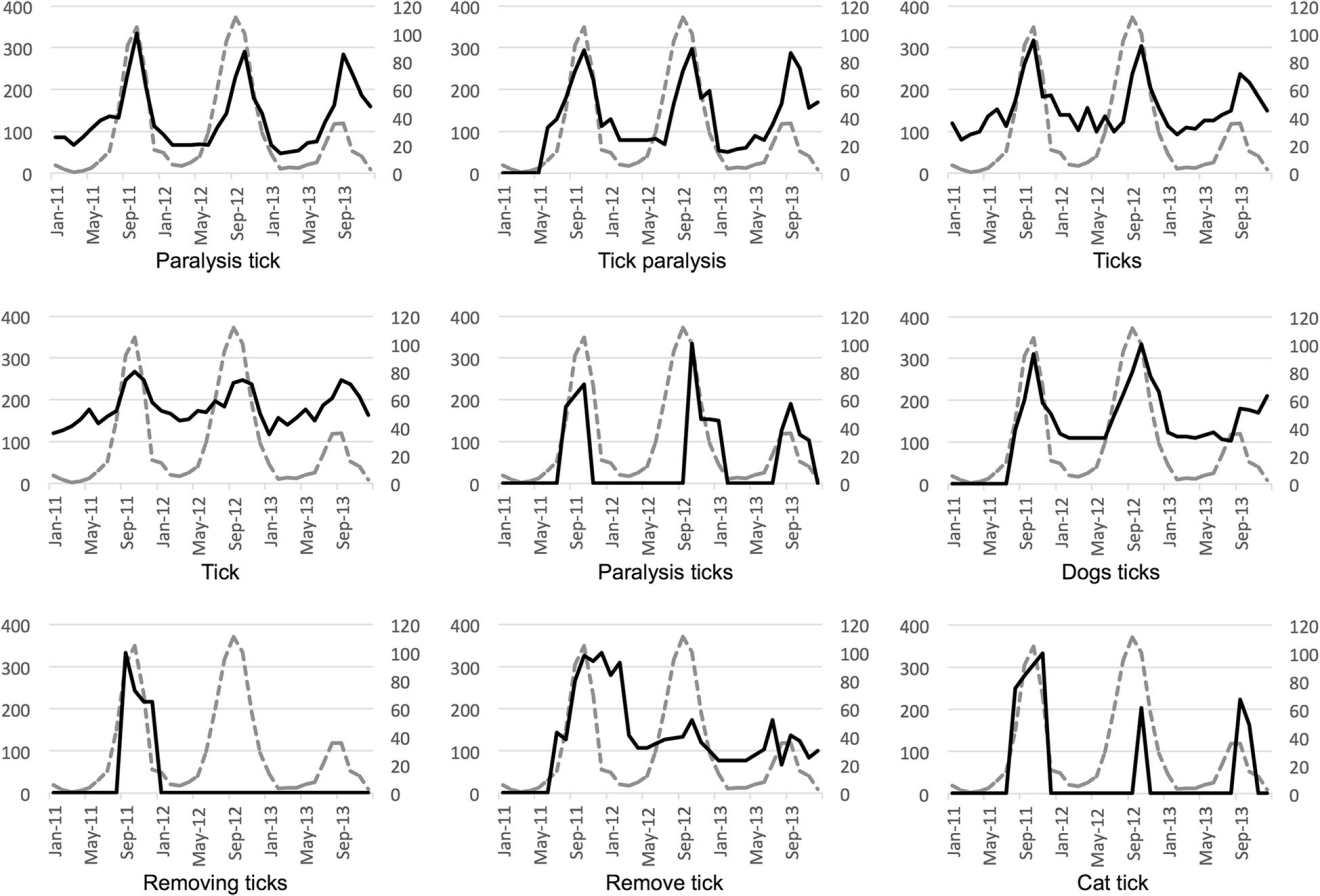
Spearman’s top ranked search terms at the state level, QLD. Gray dotted lines show the monthly notifications of tick paralysis cases in QLD, while black lines show the metrics for each of the nine search terms (on 16) that were ranked as having strongly and very strongly significant Spearman’s correlations (rho ≥ 0.6)

**Fig. 4:**
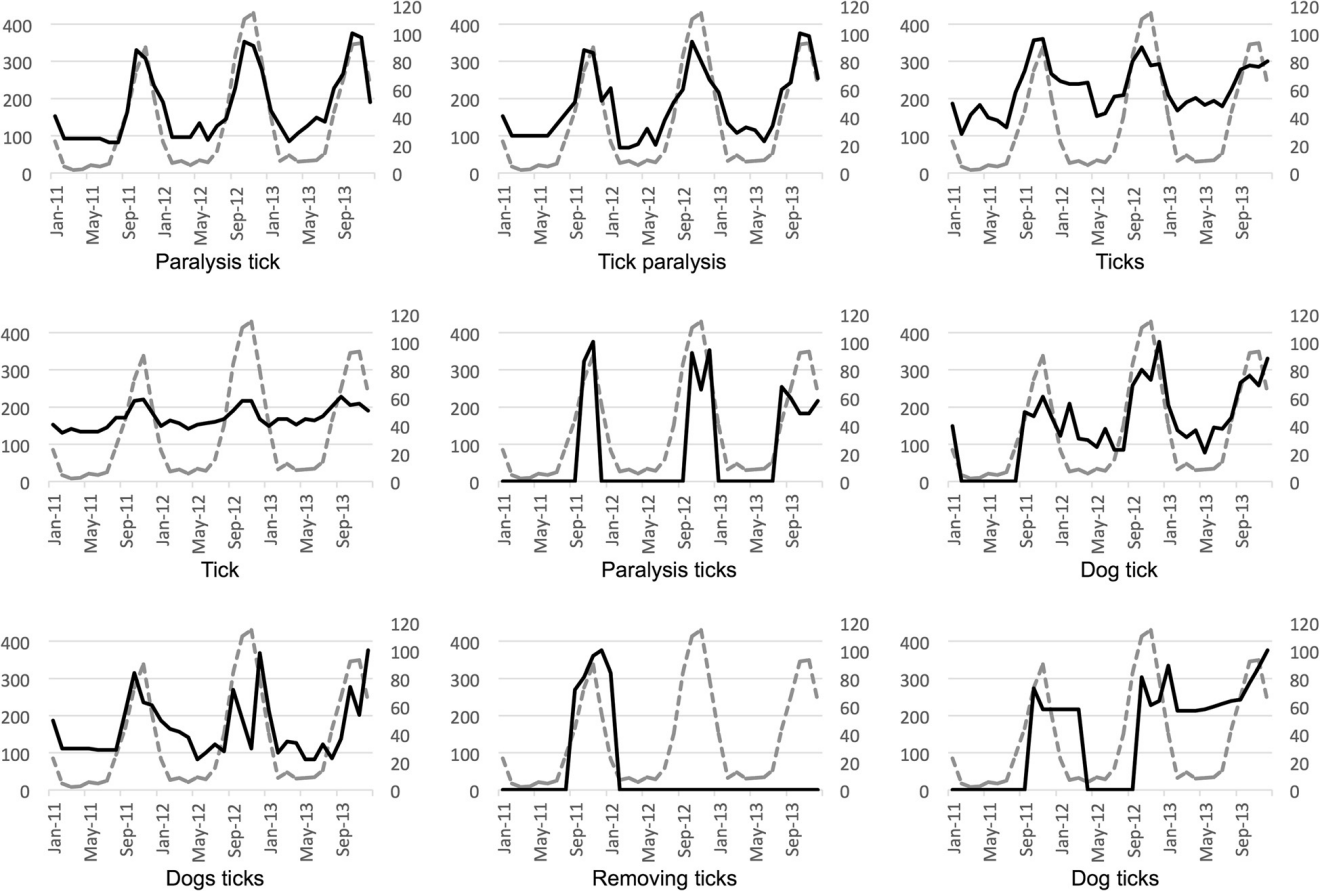
Spearman’s top ranked search terms at the state level, NSW. Gray dotted lines show the monthly notifications of tick paralysis cases in NSW, while black lines show the metrics for each of the nine search terms (on 18) that were ranked as having strongly and very strongly significant Spearman’s correlations (rho ≥ 0.6)

Our results demonstrate that all Google search terms analysed were significantly correlated to tick paralysis notifications in Australia, QLD and NSW (*P* < 0.05; Bonferroni corrected) (Table 1). Of the 24 terms analysed at the national level, 12 terms exhibited a strong correlation and five exhibited a very strong correlation with the number of tick paralysis notifications. In QLD, out of the 16 Google search terms, four presented strong correlation and five very strong correlations. In NSW, out of 18 Google search terms, four exhibited strong correlations and five exhibited very strong correlations (Table 1). The temporal relationships between Google search metrics and tick paralysis notifications for all search terms that exhibited strong or very strong correlations (rho ≥ 0.60) are presented in Figs. 2, 3 and 4.

### Cross-correlations between Google search terms and tick paralysis cases

The highest cross-correlation values, i.e. for which the two times series are best aligned, occur for lag −1, 0 or +1 (in bold, Tables 2 and 3), meaning that the peak of Google searches occur from one month before to one month after the disease notifications. At national level (Table 2), 8/17 terms peak at lag 0 (peak of Google searches coincident with notifications), 8/17 terms peak at lag +1 (Google searches occur one month after notifications) and 1/17 terms, i.e. “cats ticks”, peak at lag −1 (Google search occur one month before notifications). In QLD (Table 3), 6/9 terms peak at lag 0, while the other three peak at lag +1. In NSW (Table 3), 6/9 terms peak at lag 0, two at lag +1 and one, i.e. “removing ticks”, is not significant (for positive cross-correlations).

**Table 2:** Cross-correlation results for Australia

	(−) Values: Google searches lag notifications					(+) Values: notifications lag Google searches			
Search terms	–4	–3	–2	–1	0	1	2	3	4
Paralysis tick^b^	–0.388	–0.116	0.264	*0.652*	**0.899**	*0.86*	*0.56*	0.132	–0.261
Tick paralysis^b^	–0.381	–0.126	0.24	*0.632*	**0.871**	*0.82*	*0.511*	0.077	–0.313
Ticks^b^	–0.428	–0.207	0.18	*0.616*	**0.847**	*0.795*	*0.507*	0.187	–0.095
Tick^b^	–0.322	–0.084	0.276	*0.649*	**0.814**	*0.685*	*0.36*	0.006	–0.267
Paralysis ticks^b^	–0.363	–0.121	0.239	*0.575*	**0.786**	*0.729*	*0.49*	0.209	–0.065
Dog tick^a^	–0.474	–0.299	0.052	*0.459*	0.756	**0.829**	*0.637*	0.326	–0.021
Dogs ticks^a^	–0.554	–0.375	–0.055	*0.357*	0.641	**0.785**	*0.724*	*0.493*	0.177
Removing ticks^a^	–0.577	–0.437	–0.129	0.212	0.475	**0.547**	*0.476*	0.322	0.128
Dog ticks^a^	–0.527	–0.362	–0.07	0.286	0.607	**0.758**	*0.624*	0.346	–0.011
Remove tick^a^	–0.394	–0.284	–0.082	0.186	0.426	**0.545**	*0.473*	0.279	0.008
Cat tick^a^	–0.125	0.183	*0.44*	*0.62*	**0.692**	*0.582*	0.268	–0.087	–0.355
Paralysis tick dogs^a^	–0.405	–0.184	0.194	*0.589*	**0.864**	*0.837*	*0.579*	0.23	–0.109
Dog paralysis tick^a^	–0.44	–0.261	0.092	*0.523*	*0.836*	**0.864**	*0.562*	0.138	–0.234
Paralysis tick dog^a^	–0.429	–0.282	–0.002	*0.414*	*0.746*	**0.844**	*0.637*	0.239	–0.145
Cats ticks^a^	–0.053	0.229	*0.497*	**0.694**	*0.677*	0.472	0.177	–0.088	–0.253
Tick on dog^a^	–0.436	–0.332	–0.117	0.198	*0.515*	**0.681**	*0.649*	*0.419*	0.121
Paralysis tick symptoms^a^	–0.354	–0.134	0.222	*0.598*	**0.807**	*0.721*	*0.425*	0.065	–0.253
Confidence Limits	±0.354	±0.348	±0.342	±0.338	±0.334	±0.338	±0.342	±0.348	±0.354

The cross-correlation values are shown only for the search terms that were strongly (^a^) or very strongly (^b^) significant (Spearman’s rho ≥ 0.6). Significant positive cross-correlation values, i.e. above the upper confidence limit, are in italic, and highest values (i.e. for which the two times series are best aligned) are in bold

**Table 3:** Cross-correlation results for the states of QLD and NSW

	(−) Values: Google searches lag notifications					(+) Values: notifications lag Google searches			
Search terms	–4	–3	–2	–1	0	1	2	3	4
QLD									
Paralysis tick^b^	–0.304	–0.173	0.073	*0.445*	**0.738**	*0.705*	*0.391*	–0.018	–0.351
Tick paralysis^b^	–0.299	–0.106	0.161	*0.492*	**0.733**	*0.698*	*0.445*	0.091	–0.22
Ticks^b^	–0.282	–0.206	0.014	*0.411*	*0.719*	**0.723**	*0.453*	0.097	–0.233
Tick^b^	–0.235	–0.121	0.129	*0.470*	**0.758**	*0.724*	*0.411*	–0.007	–0.332
Paralysis ticks^a^	–0.376	–0.254	–0.017	0.3	**0.542**	*0.523*	*0.386*	0.195	–0.018
Dogs ticks^b^	–0.39	–0.205	0.1	*0.485*	*0.749*	**0.781**	*0.597*	0.32	0.047
Removing ticks^a^	–0.241	–0.208	0.009	0.292	**0.464**	*0.46*	0.286	0.054	–0.148
Remove tick^a^	–0.267	–0.149	0.067	0.293	*0.467*	**0.52**	*0.443*	0.316	0.125
Cat tick^a^	–0.268	–0.151	0.087	*0.355*	**0.581**	*0.511*	0.219	–0.111	–0.292
**NSW**									
Paralysis tick^b^	–0.312	–0.076	0.324	*0.752*	**0.941**	*0.787*	*0.409*	–0.02	–0.325
Tick paralysis^b^	–0.31	–0.019	*0.38*	*0.768*	**0.932**	*0.776*	*0.428*	–0.003	–0.326
Ticks^b^	–0.401	–0.103	0.328	*0.7*	**0.844**	*0.682*	*0.355*	0.056	–0.172
Tick^b^	–0.174	0.143	*0.512*	*0.813*	**0.853**	*0.588*	0.212	–0.098	–0.299
Paralysis ticks^a^	–0.293	–0.091	0.224	*0.621*	**0.805**	*0.664*	0.331	–0.008	–0.268
Dog tick^b^	–0.301	–0.092	0.242	*0.577*	**0.798**	*0.797*	*0.559*	–.277	–0.016
Dogs ticks^a^	–0.439	–0.25	0.049	*0.378*	*0.619*	**0.697**	*0.54*	0.258	–0.023
Removing ticks^a^	–0.335	–0.233	–0.045	0.128	0.25	0.284	0.186	–0.002	–0.157
Dog ticks^a^	–0.329	–0.254	–0.051	0.253	*0.514*	**0.632**	*0.594*	*0.434*	0.236
Confidence Limits	±0.354	±0.348	±0.342	±0.338	±0.334	±0.338	±0.342	±0.348	±0.354

The cross-correlation values are shown only for the search terms that were strongly (^a^) or very strongly (^b^) significant (Spearman’s rho ≥ 0.6). Significant positive cross-correlation values, i.e. above the upper confidence limit, are in italic, and highest values (i.e. for which the two times series are best aligned) are in bold

If the peak of Google searches and the peak of notifications occur mostly simultaneously, a positive correlation between Google searches and notifications (in italic, Table 2) generally appear earlier, i.e. before the peak of notifications. Google searches can anticipate the notifications of tick paralysis up to two months (lag −2), depending on the Google search term (Tables 2 and 3). Our results indicate that Google searches significantly lead tick paralysis notifications for 13/17 significant terms for Australia (Table 2) (two months before for two terms, i.e. “cat tick” and “cats (cat’s) ticks”; one month before for the others), 6/9 for QLD and 7/9 for NSW (Table 3). Significant search terms common to Australia and both states are: “paralysis tick”, “tick paralysis”, “ticks”, “tick” and “dogs (dog’s) ticks”. For all five terms, Google search terms lead tick paralysis notifications by one month at national and state levels, except in NSW where “tick paralysis” and “tick” lead tick paralysis notifications by two months. The other search terms that significantly lead tick paralysis notifications but are specific to national or state levels are listed in Tables 2 and 3. Our results also indicate that in Australia (Table 2), the Google search terms “removing ticks”, “dog ticks”, “remove tick” and “tick on dog” are synchronous with tick paralysis notifications (significant positive cross-correlation at lag 0). In QLD, the Google search terms “paralysis ticks”, “removing ticks” and “remove tick” are synchronous with tick paralysis notifications, whereas in NSW, the Google search term “dog ticks” only is synchronous with notifications (Table 3).

## Discussion

This study investigated the potential of Internet-based surveillance systems for the monitoring and early detection of tick paralysis in companion animals across Australia by comparing Internet search metrics with three years of notification data from the passive surveillance system *Disease WatchDog*. The analysis of these data has enabled us to identify a limited set of Google search terms of significant epidemiological importance for the monitoring and early detection of tick paralysis cases in companion animals in Australia.

Our findings are significant for three main reasons. First, previous studies in eastern Australia reported a seasonal abundance of young adult ticks during spring and early summer in QLD and NSW [6, 21, 22], coincident with a peak in tick paralysis notifications in Australia in spring, with 65.5 % of all cases being reported from September to November [2]. Our analysis of the time-series of monthly notifications of tick paralysis in dogs and cats extends current knowledge in that it suggests that the seasonal pattern differs between the eastern states of QLD and NSW; our results show that tick paralysis notifications in QLD rise and peak one month earlier compared to NSW (i.e. September-October in QLD *vs* October-November in NSW). The gap of one month that we report here between QLD and NSW could partly be explained by a temporal lag in optimal weather and environmental conditions for the proliferation of ticks between the two states [2, 23, 24].

Secondly, our study demonstrates that a tick paralysis surveillance system based on Internet search data may have great potential for the development of prevention and control strategies for the disease. We could highlight five search terms common to Australia, QLD and NSW which lag tick paralysis notifications by one or two months. Other terms were shown to be more specific to a geographic level but strongly significant, and anticipate notifications by one or two months. Five search terms were significant at the national level only, which could be related to small sample sizes at state level. Four search terms were best correlated with notifications at lag 0 at national and state levels, referring to synchronicity between Google searches and notifications. Two terms, i.e. “removing ticks” and “remove tick”, were best correlated with notifications at lag +1 for Australia and QLD but were already significant at lag 0. The use of those search terms exhibits particular importance as they can be used to develop monitoring systems, and pinpoint the need for educating pet owners about how to remove ticks and/or for the distribution of tick removal tools.

Finally, the results of this study have important public health implications in that early detection of the seasonal increase of tick paralysis in states of Australia (potentially at different times) would allow veterinarians to anticipate high-risk periods, enabling them to prepare in a timely manner as case load escalates. Access to this sort of information would also allow clinics to provide improved, more accurate, evidence-based advice/reminders to owners on risk of tick envenomation and vigilance of prophylaxis. Indeed, vigilance of prophylaxis is even more important as client compliance in prophylaxis application has been demonstrated to be poor [10]. Moreover, as this surveillance is based on automated data acquisition, it is inexpensive and may avoid the reporting bias associated to conventional passive surveillance systems. Furthermore, despite affecting mostly dogs and cats, tick paralysis has been reported in humans and livestock, and is a potentially fatal illness of importance to both human and veterinary medicine [1, 4, 6, 9, 25]. Thus, the monitoring of tick paralysis in companion animals may present positive effects not only in the control of the disease in animals, but also in humans.

Some limitations need to be considered in this study. Firstly, we only had access to monthly data on tick paralysis through the *Disease WatchDog* system, when daily or weekly reports, which are commonly used in traditional surveillance systems, could potentially reveal more fine-grained, clinically relevant, trends [16]. Secondly, we aggregated tick paralysis cases from dogs and cats due to insufficient number of cases in cats; however, in future studies data from dogs and cats should be disaggregated to enable the identification of species-specific epidemiological characteristics of tick paralysis cases. Lastly, the study analysed notifications only for the three years that had relevant available data (2011–2013) totalizing 36 months, which is a low number of observations. In our study, the time-series of *Disease WatchDog* tick paralysis notifications showed a strong decrease of online notifications within and after 2013 compared to previous years which is very likely an artefact brought about by less compliance by users of the system [26]. It is therefore important to find complimentary data sources that can be used to monitor long-term trends in the number of tick paralysis cases. Despite these shortcomings, the results of this study demonstrate clear potential for digital surveillance approaches in monitoring not just tick paralysis, but diseases of veterinary significance in general.

## Conclusion

In summary, the present study demonstrates that the monitoring and early detection of high-risk periods for tick paralysis cases in companion animals in the community could be performed through Internet-based surveillance using a limited set of Google search terms. Moreover, to our knowledge, this is the first study that demonstrates the applicability of an Internet-based surveillance approach to a disease of veterinary significance. Future work towards building of a relevant and effective real-time model of tick paralysis cases in companion animals should consider combining Google Search data identified in the present paper to other data on environmental factors such as climate time series on rainfall and temperature and physical environment time series such as landcover and landuse.
